# Dissolution of Non-cohabiting Relationships and Changes in Life Satisfaction and Mental Health

**DOI:** 10.3389/fpsyg.2022.812831

**Published:** 2022-03-04

**Authors:** Richard Preetz

**Affiliations:** SOCIUM Research Center on Inequality and Social Policy, Department of Social Sciences, Institute of Sociology, University of Bremen, Bremen, Germany

**Keywords:** non-cohabiting, living apart together, mental health, life satisfaction, dissolution, longitudinal data, adjustment

## Abstract

This study investigates how individuals’ life satisfaction and depression are affected by the dissolution of a steady non-cohabiting intimate relationship. Previous studies have focused more on the consequences of divorce and less on the influence of non-cohabiting relationships on the well-being of the individual. The data for this study were taken from pairfam, a large-scale German panel survey, and were used to estimate fixed-effects panel regression models and impact functions to identify the overall effect of dissolution and trajectories after separation. The study sample comprised 2,631 individuals who were observed over the course of 11,219 partnership years. Based on the results of this study, three main findings were reported. First, the dissolution of a non-cohabiting relationship led to a significant decline in mental health and life satisfaction. Second, the trajectories after dissolution suggest that the decline was only temporary, showing readjustment after 1 year. Third, gender differences were identified, suggesting worse consequences for men who experienced a significant decline in both dimensions and did not readjust in life satisfaction until several years after the dissolution. For women, decreases were only found for life satisfaction, but quick readjustments were observed.

## Introduction

The dissolution of an intimate relationship is one of the most stressful life-course events (Lorenz et al., 2006; Mather et al., 2014; Hald et al., 2020). During the dissolution, many changes occur within a relatively short period of time, including novel feelings of anger and sadness, a loss of companionship and social support, and adaptation to a single lifestyle (Amato, 2000, 2010). Adopting assumptions from stress theory, it can be indicated that this concentration of potential stressors can adversely affect individuals’ health and well-being (Pearlin et al., 2005; Pearlin, 2010). Numerous studies have identified the negative effects of dissolution on mental and physical health as well as on psychological well-being and life satisfaction (Hewitt and Turrell, 2011; Hewitt et al., 2012; Sbarra et al., 2014; Sbarra, 2015; Leopold and Kalmijn, 2016; Kalmijn, 2017; Leopold, 2018; Hald et al., 2020; Sander et al., 2020). However, most of these studies had focused only on the dissolution of marriages. While divorce remains undoubtedly a major life-course transition, types of partnerships and living arrangements have become more diverse in recent decades (Feldhaus and Preetz, 2021). Shifts in the occurrence, trajectories, and dynamics of intimate relationships and living arrangements have been observed. Falling marriage rates, rising divorce rates, and higher rates of extramarital births have resulted in “the retreat of marriage” and the “de-institutionalization of marriage” (Cherlin, 2004; Sassler and Lichter, 2020). A key characteristic of these ongoing demographic trends is the growth in the number of couples who cohabitate without marriage or are living in separate households (Hiekel et al., 2014; Liefbroer et al., 2015).

This study focuses on the dissolution of living apart together or non-cohabiting intimate relationships. Cross-sectional data had indicated that the prevalence rates of couples living in separate households ranged from approximately 6 to 10% in Australia (Reimondos et al., 2011), Canada (Milan and Peters, 2003), the United States (Strohm et al., 2009), the United Kingdom (Coulter and Hu, 2017), France (Regnier-Loilier et al., 2009), Germany (Asendorpf, 2008), and Eastern Europe (Liefbroer et al., 2015). From a longitudinal perspective, being a member of a non-cohabiting relationship is a standard sequence for nearly every intimate relationship, especially at the beginning (Peter et al., 2015; Pasteels et al., 2017). Only a small number of couples remain in separate households for over many years, with the majority breaking up or beginning to cohabit, making living apart together a highly transitory type of partnership (Dorbritz and Naderi, 2012; Schnor, 2015; Régnier-Loilier, 2016; Bastin, 2019; Wagner et al., 2019). Thus, unlike divorce, most people experience the dissolution of a non-cohabiting intimate relationship. However, only a few studies have examined the consequences of dissolution for individuals’ well-being and health (Rhoades et al., 2011; Mirsu-Paun and Oliver, 2017; Norona and Olmstead, 2017; Waterman et al., 2017).

Being in an intimate relationship is associated with benefits for psychological and physiological health and well-being. A romantic partner is expected to provide resources that enhance well-being, including companionship, social and emotional support, love, and sexual involvement (Dush and Amato, 2005; Soons et al., 2009; Amato, 2010; Braithwaite et al., 2010; Rhoades et al., 2011). Most studies focused on marriage have suggested that married people have better overall health and less psychological distress and are less often depressed (Kalmijn, 2017). The few studies that explicitly focus on non-cohabiting relationships have found higher levels of subjective well-being and fewer mental health problems compared with single individuals (Dush and Amato, 2005; Braithwaite et al., 2010). People in non-cohabiting relationships had reported high levels of emotional and instrumental support between partners, low levels of strain, and high levels of personal autonomy (Strohm et al., 2009; Duncan et al., 2014; Lewin, 2017). Two recent longitudinal studies had investigated the influence of transitions in an intimate relationship on the individual’s subjective well-being and mental health. Both studies had found separate effects for transition into a non-cohabiting relationship, cohabitation, and marriage, with increases in both dimensions (Soons et al., 2009; Rapp and Stauder, 2020).

The loss of factors promoting relationship-related health and well-being as a consequence of dissolution can negatively affect individuals’ health and well-being (Amato, 2010). Previous results had suggested that the loss of benefits due to dissolution has a 2-fold or 3-fold greater impact than the gain of benefits from the beginning of marriage (Kalmijn, 2017). Amato (2000, 2010) posits a divorce-stress-adjustment perspective to explain the negative outcomes of divorce on well-being and health. Although this model was initially developed in relation to divorce, it can be adapted to the dissolution of nonmarital relationships. The end of an intimate relationship is generally conceived more as a process than an event. Typically, the uncoupling process leads to numerous events within a relatively short period of time, making it a stressful experience. Stressors, such as the loss of love, companionship, and social and emotional support, along with the new tasks of adjusting to living alone, informing families and friends, and possibly finding a new partner, increase the risk of negative outcomes in health and well-being (Amato, 2000; Leopold and Kalmijn, 2016; Tosi and van den Broek, 2020). Embedded within the divorce-stress-adjustment framework are two contrary models that determine whether the negative outcomes of dissolutions are only temporary or persist more or less indefinitely (Amato, 2000, 2010; Raley and Sweeney, 2020; Whisman et al., 2022). First, a crisis model indicates that the effects of relationship dissolution only persist for the short term. After immediate decreases in health and well-being in relation to dissolution, people often adapt to their new daily living routines after a sufficient amount of time, and their levels of well-being and health return to their baseline levels prior to dissolution. Assumptions from set-point theories support this idea of an adaptation effect. It assumes that an individual’s subjective well-being has a certain baseline level, determined by genes and stable personality factors, which fluctuates only temporarily around a stable set point. In response to major life events, short term increases or decreases of well-being often show a rapid adjustment to baseline levels (Diener et al., 2006; Anusic et al., 2014). However, the chronic strain model assumes persistent strains following relationship dissolution. Stressful changes in life circumstances could accumulate over time, resulting in permanently lower levels of health and well-being, which may affect the ability to cope with other stressful life events (Lorenz et al., 2006; Tosi and van den Broek, 2020).

This paper investigates how individuals’ life satisfaction and mental health are affected by the dissolution of a non-cohabiting relationship. It contributes to the literature in the following three ways. First, although previous studies often focus on cohabiting and marital relationships, several findings from non-cohabiting dating relationships suggested increases in depression, sadness, and anger after a breakup (Monroe et al., 1999; Sbarra and Emery, 2005; Sbarra, 2006; Reyes-Rodríguez et al., 2013; Liang and Horn, 2020). This study contributes to the state of research by analyzing steady non-cohabiting relationships and foregrounds steady relationships with lower levels of institutionalization. While previous studies often used relatively small sample sizes with short time intervals, this study uses large-scale panel data over several years to uncover the consequences and trajectories of well-being and mental health after the relationship dissolution. Second, using large-scale panel data allows the permanency of changes in well-being and mental health to be identified. Previous findings on divorce have provided evidence for the crisis model (Hetherington, 2003; Wade and Pevalin, 2004; Hughes and Waite, 2009; Leopold and Kalmijn, 2016; Kalmijn, 2017; Leopold, 2018), indicating that people experience a decline in life satisfaction and mental health immediately after the end of a relationship but adjust over the next 2–3 years and return to their levels prior to the divorce. Results from dating relationships also indicated a recovery in depression, sadness, and anger after a breakup (Sbarra, 2006; Verhallen et al., 2021). However, due to the relatively short time intervals in these previous studies, findings of whether adaptation processes follow the same pattern over a long time in the dissolution of steady non-cohabiting relationships than in higher institutionalized relationships are rare. Third, gender differences in life satisfaction and mental health trajectories are analyzed. Several studies have shown that men are more vulnerable to the negative effects of divorce, including a greater decline in life satisfaction and mental health (Andreß and Bröckel, 2007; Blekesaune, 2008; Shor et al., 2012; Symoens et al., 2014; Leopold, 2018). Furthermore, research on gender differences in relation to the dissolution of non-cohabiting relationships is limited.

## Materials and Methods

The following analyses are based on waves 2–11 (2009/10–2018/19) of the Panel Analysis of Intimate Relationships and Family Dynamics (pairfam; Huinink et al., 2011; Brüderl et al., 2021). Pairfam is an annual interdisciplinary panel survey of randomly selected men and women in Germany. Beginning in 2008 with 12,402 participants from three birth cohorts (1971–93; 1981–83; and 1991–93), pairfam is well suited to identify the development of non-cohabiting relationships over many years.

### Participants

Respondents in a steady non-cohabiting relationship were identified by answering the following survey questions: (1) “Do you have a steady relationship at the moment?” and (2) “Do you live together with this partner in the same dwelling?” To analyze the effects of relationship dissolution on life satisfaction and mental health, all periods of being in a non-cohabiting relationship were considered. To identify partnership dissolutions, pairfam used an innovative type of an event history calendar covering the period between the previous and current survey interview (Brüderl et al., 2017). Based on the information from the previous wave, a partnership calendar was shown to the respondents including their name, gender, and date of birth. Then, the respondents had to specify whether they were still in an intimate relationship with this partner or not. If otherwise, they had to specify the month of the dissolution. Individuals were followed up during the dissolution process for as long as possible, that is, until the time of the last interview or the beginning of a new relationship. During the observation period, a person may have multiple episodes of different non-cohabiting relationships. New ID variables were obtained for each individual to recognize each new relationship separately in the statistical models. If a respondent moved in with their partner before separation or before the most recent interview, these episodes were treated as censored. Additional sensitive analyses showed no substantial changes for the effects of dissolution if these episodes were excluded. Note that all data were collected prior to the outbreak of the coronavirus pandemic. Although a further panel wave with data collected during the pandemic was present, its data were not considered due to selectivity issues associated with the change of the interview mode (Bozoyan et al., 2021). The final sample included 2,631 individuals with 3,206 non-cohabiting partnerships that were observed over 11,219 partnership years. Moreover, 1,609 dissolution events were recorded. Considering the cohort design of pairfam, the sample was relatively young, ranging from 18 to 47 years old (*M* = 26.41, *SD* = 7.73). The percentage of women was 51.05%. The mean length of the relationships before they ended was 2.05 years (*SD* = 2.10) with a median of 1.5 years. Table 1 shows the descriptive information of all variables and sample characteristics.

**Table 1:** Descriptive data.

	Overall	Women	Men
Depression (1–4)	1.79	1.84	1.73
Life satisfaction (0–10)	7.53	7.46	7.61
Unemployed	6.03%	6.11%	5.94%
Education finished	26.91%	27.69%	26.09%
Having children	1.94%	2.67%	1.18%
Aged 18–24	49.55%	49.64%	49.45%
Aged 25–29	22.03%	19.49%	24.67%
Aged 30–35	13.01%	12.68%	13.36%
Aged 36–41	8.06%	9.08%	6.99%
Aged 42–47	7.35%	9.11%	5.52%

*Data: Pairfam waves 2–11; own calculations*.

### Measures

An event dummy was created to detect the overall effects of the dissolution of a non-cohabiting relationship with the following values: 0 = no dissolution and 1 = dissolution. To investigate whether changes due to dissolution are only short term or permanent, the event dummy was more differentiated and combined with the time since dissolution. This new event-centered variable captures the year of the end of the relationship. Moreover, the following years were marked, resulting in a variable with the following values: 1 = year of the dissolution; 2 = 1 year after the dissolution; 3 = 2 years after the dissolution; and 4 = 3 or more years after the dissolution. The reference category 0 includes all person-years during the relationship before the dissolution occurred.

Life satisfaction was measured using the following question: “All in all, how satisfied are you with your life at the moment?” The answers ranged from 0 (“very dissatisfied”) to 10 (“very satisfied”). The mean value over all the observation periods was 7.53 (*SD* = 1.64).

Mental health was measured as levels of depression using the State-Trait-Depression Scale (10 items; STDS Form Y-2; Spaderna et al., 2002). This scale consists of five items assessing negative mood and five items assessing positive mood. The sample items include “My mood is melancholy” or “I feel good,” with response values ranging from 1 (“almost never”) to 4 (“almost always”). The mean value over all the observation periods was 1.79 (*SD* = 0.51).

Because fixed-effects models were used for the statistical analyses (see below), time-constant variables were unnecessary. The controls were time varying, including being unemployed, in education, and having children, as well as age intervals (18–23; 24–29; 30–35; 36–41; and 42–47). These variables are relevant for well-being and mental health and are related to the dissolution of relationships (Lois and Lois, 2012; Gebel and Voßemer, 2014; Krapf, 2018; Wagner et al., 2019; Nomaguchi and Milkie, 2020).

### Analytical Plan

Fixed-effects panel regression models were used to investigate the effects of relationship dissolution on life satisfaction and mental health. Standard between regressions that model the effect of divorce/dissolution on well-being and health may be biased because it is impossible to control for all relevant potential confounders, whether observed or unobserved (Allison, 2009; Brüderl and Ludwig, 2014). Fixed-effects models are helpful for this limitation because they use within-person changes induced by a treatment variable instead of inferring effects from group comparisons (Amato, 2010). The main advantage of this method is that its focus on persons’ within-variation enables unobserved heterogeneity to be controlled for, thus preventing confounding effects that could result from persons’ between-variation. In more detail, instead of conducting a between-variation comparison of the individuals who were in a non-cohabiting relationship or are single, the effect of dissolution on well-being and mental health was identified based on the differences between the individuals’ levels before and after the end of an intimate relationship. Stable characteristics or heterogeneity between the individuals no longer influence the effects of dissolution on well-being and mental health. Furthermore, it was established that selection effects did not bias the results by eliminating the possibility that changes in the outcomes after the event occurred could have been due to average differences in the baseline outcome levels prior to the event (Anusic et al., 2014). To model the progress of life satisfaction and mental health after dissolution, fixed-effects impact functions were used (Andreß et al., 2013; Ludwig and Brüderl, 2021). These allow the time path of an effect to be modeled based on an event-centered variable (see above). Differences in individuals’ within-variation prior to the dissolution and different time points after dissolution enabled adjustments in life satisfaction and mental health, especially if effects persist permanently. For fixed-effects panel regression models, controlling for time or period effects as an independent variable is essential if possible period trends within the control group of those who did not experience the treatment were to be considered. Previous findings suggest that life satisfaction and mental health change over the life course (Pavot et al., 1998; Pratt and Brody, 2014; Gnambs and Buntins, 2017; Weinberger et al., 2018). Thus, treatment effects could be overestimated if the period trends are not controlled for. These general time trends were considered to uncover the effects of relationship dissolution by including period dummies for every panel wave and the respondents’ age. All models were estimated separately for men and women to establish life satisfaction and mental health trajectories after the end of an intimate relationship.

## Results

The results of several fixed-effects panel regression models are presented separately below to identify outcomes in mental health and life satisfaction. At first, the overall effects of the dissolution of a non-cohabiting relationship are presented separately for men and women. Then, results from the fixed-effects impact functions are presented in graphic form to show the trajectories for both outcomes after the end of the intimate relationship.

### Mental Health

**Figure 1:**
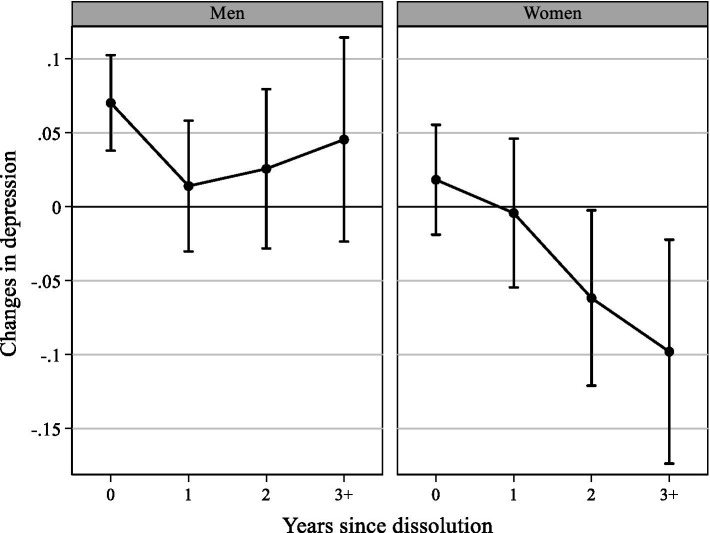
Fixed-effects panel regression impact functions for changes in depression separated by sex. Results reveal the changes in depression after relationship dissolution compared to average levels of depression before the dissolution; the value 0 on the x-axis marks the first observation point immediately after dissolution; the y-axis shows unstandardized regression coefficients from fixed-effects panel regression impact functions with 95% CI and panel robust standard errors; and data: pairfam waves 2–11; own calculation.

Beginning with changes in individuals’ depression, Table 2 shows the overall effect of the dissolution of a non-cohabiting partnership of *β* = 0.035 (*p* = 0.004; Model 1). Overall, individuals’ depression increased at the end of an intimate relationship. Differentiation shows that the overall effect depended on the negative consequences for men. Their depression significantly increased by a factor of *β* = 0.056 (*p* = 0.001), indicating that men experienced increased depression after the dissolution of a non-cohabiting intimate relationship (Model 2). The effect for women was also positive but not significant (*p* = 0.505; Model 3). Regarding the trajectories of depression during the years after the dissolution, Figure 1 shows the results for men and women. On the x-axis, the zero value marks the first observation immediately following the dissolution; 1 indicates 1 year after the dissolution and so on. For men, the results suggested an increase in depression immediately after the end of the relationship, followed by a quick readjustment 1 year later. Immediately after separation, men experienced an increase of *β* = 0.070 (*p* = 0.000) relative to their average depression before the dissolution (Figure 1). Further effects found in the following years were not significant, indicating no difference compared with the pre-dissolution levels. Conversely, women experienced no significant changes in depression in the short term after the end of their non-cohabiting relationship. Instead, they showed a decrease in depression compared with the pre-dissolution levels 2 or more years after the dissolution. Note that the observation period ended at the time of the last interview or at the beginning of a new relationship. Becoming less depressed could be the result of anticipation effects that appear as the respondent becomes acquainted with a potential new partner.

**Table 2:** Fixed-effects panel regression for dissolution on depression separated by sex.

	(1)	(2)	(3)
	Overall	Men	Women
Dissolution	0.0350^***^	0.0560^***^	0.0120
	(0.0121)	(0.0162)	(0.0180)
Unemployed	0.108^***^	0.116^***^	0.101^***^
	(0.0236)	(0.0315)	(0.0351)
Completed education	0.0287^**^	0.0216	0.0337^*^
	(0.0125)	(0.0172)	(0.0179)
Having children	0.0365	0.0968	−0.0105
	(0.0424)	(0.0680)	(0.0545)
Aged 24–29	0.00936	0.0458^*^	−0.0352
	(0.0187)	(0.0251)	(0.0277)
Aged 30–35	0.0231	0.0667^*^	−0.0255
	(0.0312)	(0.0400)	(0.0485)
Aged 36–41	0.00833	1.75e − 05	0.00952
	(0.0489)	(0.0629)	(0.0746)
Aged 42–47	−0.0285	−0.0639	−0.0167
	(0.0590)	(0.0807)	(0.0876)
Constant	1.638^***^	1.603^***^	1.670^***^
	(0.0208)	(0.0280)	(0.0310)
Periods controlled	Yes	Yes	Yes
Number of periods	11,219	5,492	5,727
Number of persons	2,631	1,266	1,365
Number of partnerships	3,206	1,540	1,666
Number of events	1,609	837	772
R_within_	0.031	0.043	0.028

*Unstandardized regression coefficients; Panel robust standard errors in parentheses*.

* p < 0.1;

** *p < 0.05;*

*** *p < 0.01. Data: Pairfam waves 2–11; own calculation*.

### Life Satisfaction

**Figure 2:**
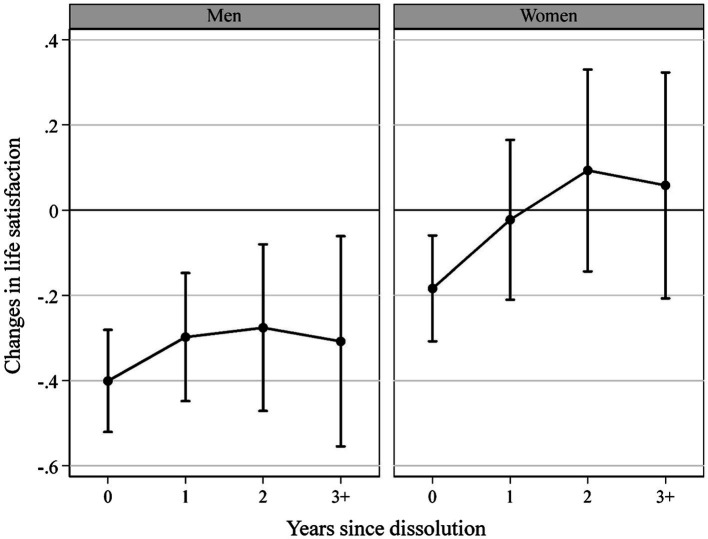
Fixed-effects panel regression impact functions for changes in life satisfaction separated by sex. Results reveal the changes in life satisfaction after relationship dissolution compared to average levels of life satisfaction before the dissolution; the value 0 on the x-axis marks the first observation point immediately after dissolution; the y-axis shows unstandardized regression coefficients from fixed-effects panel regression impact functions with 95% CI and panel robust standard errors; and data: pairfam waves 2–11; own calculation.

Table 3 shows the effects of the changes in life satisfaction. Overall, the dissolution of a non-cohabiting intimate relationship decreased life satisfaction by *β* = −0.026 (*p* = 0.000; Model 1). Men and women separately showed similar effects. Both experienced a decline in the overall level of life satisfaction (men *β* = −0.376 *p* = 0.000; women *β* = −0.139 *p* = 0.023). However, they exhibited differentiated trajectories of life satisfaction. Men’s life satisfaction declined immediately after the end of their relationship by *β* = −0.401 (*p* = 0.000; Figure 2). Although the effect was reduced over the following years, men’s life satisfaction remained significantly lower than the pre-dissolution levels. Unlike the trajectory of depression, no readjustment for men’s life satisfaction was observed. At the first observation, women experienced a decrease in life satisfaction after their relationship ended by *β* = −0.184 (*p* = 0.004; Figure 2). However, unlike men, they experienced adjustment in life satisfaction immediately in the following year. Furthermore, the levels of life satisfaction from 1 year after the dissolution did not differ from the pre-dissolution levels.

**Table 3:** Fixed-effects panel regression for dissolution on life satisfaction separated by sex.

	(1)	(2)	(3)
	Overall	Men	Women
Dissolution	−0.261^***^	−0.376^***^	−0.139^**^
	(0.0416)	(0.0577)	(0.0607)
Unemployed	−0.594^***^	−0.675^***^	−0.520^***^
	(0.0921)	(0.118)	(0.143)
Completed education	−0.00113	−0.0416	0.0385
	(0.0439)	(0.0618)	(0.0622)
Having children	−0.176	−0.120	−0.160
	(0.209)	(0.267)	(0.292)
Aged 24–29	−0.0241	−0.0237	−0.00584
	(0.0645)	(0.0909)	(0.0917)
Aged 30–35	−0.0675	−0.141	0.0255
	(0.114)	(0.156)	(0.167)
Aged 36–41	−0.226	0.203	−0.571^*^
	(0.217)	(0.292)	(0.308)
Aged 42–47	−0.0304	0.342	−0.349
	(0.253)	(0.352)	(0.354)
Constant	7.998^***^	7.948^***^	8.064^***^
	(0.0728)	(0.0973)	(0.109)
Periods controlled	Yes	Yes	Yes
Number of periods	11,219	5,492	5,727
Number of persons	2,631	1,266	1,365
Number of partnerships	3,206	1,540	1,666
Number of events	1,609	837	772
R_within_	0.034	0.051	0.027

*Unstandardized regression coefficients; Panel robust standard errors in parentheses*.

* *p < 0.1;*

** *p < 0.05;*

*** *p < 0.01*.

*Data: Pairfam waves 2–11; own calculation*.

## Discussion

Having a romantic partner and an intimate relationship have a favorable effect on the individual’s mental health and subjective well-being. Romantic partners benefit from resources that enhance health and well-being, such as companionship, social and emotional support, love, and sexual involvement. This study investigated the consequences of relationship dissolution of steady non-cohabiting relationships on the individuals’ life satisfaction and mental health. Losing the potential benefits from being in an intimate relationship because of separation is associated with a decline in psychological and subjective well-being. Using large-scale panel data from Germany, the overall effects of dissolution on life satisfaction and depression were estimated. The trajectories of the outcomes following the end of the relationship were modeled and analyzed separately for men and women. The use of fixed-effects panel regression models allowed to estimate individuals’ within-variation to be assessed prior to and after the end of their intimate relationships.

The results revealed three main findings. First, the dissolution of a non-cohabiting relationship led to a significant decline in the individuals’ mental health and life satisfaction. The respondents reported higher levels of depression and lower life satisfaction after the end of their partnerships relative to pre-dissolution levels. Most studies had focused more on the negative outcomes of divorce from marriage and less on intimate couples who live in separate households (Soons et al., 2009; Rhoades et al., 2011; Rapp and Stauder, 2020). This study used large-scale panel data and confirmed previous findings from non-cohabiting dating relationships to illustrate the importance of non-cohabiting relationships for mental health and life satisfaction trajectories. Adverse dissolution outcomes were often associated with stressors related to living in the same household, such as finding a new accommodation or the division of goods (Amato, 2000; Leopold and Kalmijn, 2016; Tosi and van den Broek, 2020). However, the results of this study suggest that even partnerships with lower levels of institutionalization affect the loss of mental health and life satisfaction. Findings from cross-sectional studies have suggested higher levels of subjective well-being and mental health and low levels of strain as well as higher emotional and instrumental support between partners in non-cohabiting relationships compared with being single. The loss of these lower institutionalized benefits along with the loss of a relationship can be attributed to unfavorable outcomes.

Second, drawing on competing assumptions from crisis and chronic strain models, the results of this study suggest that a decline in mental health and life satisfaction should be only temporary. Research on the dissolution of marriage showed a similar pattern for adjustment, confirming the assumption of dissolution as a temporary crisis (Hetherington, 2003; Wade and Pevalin, 2004; Leopold and Kalmijn, 2016; Kalmijn, 2017; Loter et al., 2019). Readjustment followed and made up for increased depression as well as decreased life satisfaction that occurred immediately after dissolution in the following years. A more detailed comparison between the different types of partnerships suggests some evidence for a more rapid adjustment after the dissolution of a non-cohabiting partnership. While most changes in depression and life satisfaction readjust 1 year after the end of the partnership in non-cohabiting relationships, the process of recovery takes a little longer for cohabiting or married partnerships. Findings from Germany and Swiss had indicated that life satisfaction and mental health readjust after 2–5 years after the divorce or separation with weaker effects for unmarried cohabitation (Leopold and Kalmijn, 2016; Kalmijn, 2017). Other findings had suggested faster adjustments with recovery 1 year after the divorce leading to an inconsistent knowledge of temporal patterns following divorce (Wade and Pevalin, 2004; Leopold, 2018). However, the process of readjustment in mental health and life satisfaction after dissolution may be a universal force unrelated to the grade of relationship institutionalization and whether couples lived in the same household prior to the dissolution (Clark et al., 2008).

Third, gender differences in relation to the outcomes and their pathways after dissolution were observed. Although the overall effects for depression and life satisfaction were significant, separate models showed no significant dissolution effects for women’s depression. Only men reported significantly higher levels of depression after the end of their partnerships. Usually, a decline in life satisfaction and an increase in depression adjust over time, but men were found to be more affected in the long term than women. In the present study, men’s life satisfaction declined and did not adjust after the end of a non-cohabiting intimate relationship. Even 3 years after the separation, the levels of life satisfaction were significantly lower than the pre-dissolution values. Studies on divorce have suggested that men are more vulnerable to the adverse effects of divorce than women (Hewitt and Turrell, 2011; Shor et al., 2012; Leopold, 2018). However, men may benefit more from the resources gained by a romantic partner, like social and emotional support, or companionship. Thus, separation puts them at a higher risk of declining life satisfaction and mental health. Further findings suggest that it is difficult for men to adjust to a single lifestyle. They report greater feelings of loneliness, social isolation, and lower satisfaction with singlehood than women (Dykstra and Fokkema, 2007; Symoens et al., 2014; Ochnik and Slonim, 2020; Kislev, 2021).

### Limitations and Future Research

This study is a step further in understanding the consequences of the dissolution of non-cohabiting partnerships on individuals’ mental health and well-being. The results showed similar patterns in adjustment and gender differences to the effects of divorce. Thus, considering couples who do not live in the same household is important in understanding the individuals’ trajectories of well-being. However, this study has several limitations. First, the sample is relatively young, with most individuals under the age of 30. Previous research had shown that non-cohabitating relationships are heterogeneous and relatively ambiguous depending on the individuals’ life-course position (Regnier-Loilier et al., 2009; Coulter and Hu, 2017; Pasteels et al., 2017). In young adulthood, non-cohabiting often serves as a prelude to cohabitation and marriage. Most couples live involuntary in separate households due to different life-course constraints and intend to cohabit within the next years (Liefbroer et al., 2015). On the other hand, for older adults, living in separate households serves more as a stable alternative type of partnership influenced more by choice than constraints (Connidis et al., 2017; Wu, 2019; Mauritz and Wagner, 2021). Future studies should focus on dissolution outcomes for older non-cohabiting couples to explain the process of adjustment or determine if dissolution is a temporary crisis or chronic strain. A cross-sectional study found no differences in the depression levels between older individuals in non-cohabiting partnerships and singles (Wu, 2019). For gray divorce, studies from the United Kingdom had suggested similar temporal patterns for depression with increasing levels immediately after the divorce and readjustment in the following years (Tosi and van den Broek, 2020). Although the number of studies on non-cohabiting couples in older adulthood is growing, longitudinal findings for the consequences of dissolution are still limited.

Potential mediators were not considered in the investigation of further differences in the trajectories of mental health and life satisfaction, except for gender differences. For example, the effects of dissolution may differ for those who initiated the separation. Moreover, the individuals’ partnership history may play an important role because the effects of dissolution may be different in the case of a first-ever or higher-order separation. Studies on divorce had emphasized the role of children as a mediator in dissolution outcomes. Individuals with children have been found to experience a sharper decline in life satisfaction than childless men and women (Leopold and Kalmijn, 2016). For mental health, no general difference in divorce outcomes was observed between individuals with and without children. However, those with young children tend to experience the largest and long-lasting decline in mental health (Loter et al., 2019). Since the number of individuals with children in this study is too low to conduct a differentiated analysis, further studies are needed to investigate possible differences due to the presence of children. Living in separate households is often chosen in the context of post-union after a divorce, marking the start of a new stepfamily constellation. Findings from Germany showed that the majority of single mothers with non-cohabiting relationships experienced a transition in the first 3 years, leading to the question about the effects of dissolution on parents and children (Bastin, 2019).

Furthermore, although the results suggest similar patterns of dissolution effects to those observed after the end of a marriage, no direct comparison has been tested. Future studies should estimate and compare dissolutions from different types of relationships to compare temporal patterns. Previous findings suggest that individuals anticipate upcoming major life events (Yap et al., 2012; Anusic et al., 2014). Life satisfaction and depression may not only change after dissolution but immediately before the end of the relationship. Rising conflicts or the experience of internal or external shocks may lead to a significant decline in the levels of well-being and mental health that could result in dissolution. The investigation of anticipation effects is not possible in this study because the average length of the relationship before the dissolution is relatively short compared to annual survey intervals. Separating the few pre-dissolution time intervals to analyze the anticipation effects would result in problems with selectivity due to the low share of non-cohabiting couples who were staying together for many years without a transition. Adaptation and anticipation processes may occur over shorter time intervals in non-cohabiting partnerships. Future studies could also use panel data with shorter time intervals between interviews to examine more finely differentiated trajectories.

## Data Availability Statement

Publicly available datasets were analyzed in this study. This data can be found at the German Family Panel (pairfam). GESIS Data Archive, Cologne. ZA5678 Data file Version 12.0.0, https://doi.org/10.4232/pairfam.5678.12.0.0.

## Author Contributions

The author confirms being the sole contributor of this work and has approved it for publication.

## Funding

This research is funded by the Deutsche Forschungsgemeinschaft (DFG, German Research Foundation)—Project number 165713635—Beziehungs- und Familienentwicklungspanel (pairfam).

## Conflict of Interest

The author declares that the research was conducted in the absence of any commercial or financial relationships that could be construed as a potential conflict of interest.

## Publisher’s Note

All claims expressed in this article are solely those of the authors and do not necessarily represent those of their affiliated organizations, or those of the publisher, the editors and the reviewers. Any product that may be evaluated in this article, or claim that may be made by its manufacturer, is not guaranteed or endorsed by the publisher.
